# Screening and validation of optimal miRNA reference genes in different developing stages and tissues of *Lilium henryi* Baker

**DOI:** 10.1038/s41598-024-51562-1

**Published:** 2024-01-17

**Authors:** Ge Jin, Xiuhai Zhang, Shiyin Yu, Yunpeng Du, Meixian Wang, Chunli Zhao, Mingfang Zhang

**Affiliations:** 1 Beijing Academy of Agriculture and Forestry Sciences, Key Laboratory of Urban Agriculture (North China), Ministry of Agriculture and Rural Affairs, Beijing, 100097 China; 2 College of Forestry and Grassland Science, Jilin Agricultural University, Changchun, 130118 China; 3 College of Landscape Achitecture, Beijing Forestry University, Beijing, 100097 China

**Keywords:** Gene expression analysis, miRNAs

## Abstract

Dynamic miRNA detection using the qRT-PCR technique requires appropriate reference genes to ensure data reliability. Previous studies have screened internal reference genes in plants during embryonic development and various stress treatment, involving relatively few tissues and organs. There is no relevant miRNA study in *Lilium henryi* Baker and limited research on the optimal miRNA reference genes in lilies, such as 5S, 18S, U6 and Actin. Twelve genes were selected as candidate reference genes whose expression stability was analyzed in petals at different developmental stages and other tissues using various algorithms, such as geNorm, NormFinder, BestKeeper, and Delta CT. The results revealed that the optimal combination of reference genes for *Lilium henryi* Baker petals at different developmental stages was osa-miR166m and osa-miR166a-3p, while that for different tissues of *Lilium henryi* Baker was osa-miR166g-3p and osa-miR166a-3p.Four important genes related to growth and development regulation, namely, osa-miR156a, osa-miR395b, osa-miR396a-3p, and osa-miR396a-5p, were selected for validation. The findings of the present study could contribute to future investigations onmiRNA expression and the related functions in *Lilium henryi* Baker while providing important references for the normalization of the miRNA expression in other varieties of lily.

## Introduction

Lilies (*Lilium* spp.) are considered among the most important floral crops worldwide^1,2^. Lilies are recognized for their diverse flower colors and forms and are, therefore, widely used in garden beautification and cut flower markets^3–5^. The growth and development of lilies involve a series of biochemical processes in which miRNAs (microRNAs) play important roles, for instance, in cell proliferation, cell differentiation, growth, development, and response to external environmental stimuli^6–8^. Plant miRNAs were identified in *Arabidopsis* and *Oryza sativa* in 2002^9^, and since then, hundreds of similar small RNA molecules, which are collectively referred to as miRNAs, have been identified in several model organisms and cells. The above studies have revealed the complexity of gene regulatory networks in plants, due to which miRNAs have become a research hotspot in recent years.

An unstable expression of a reference gene could lead to biased outcomes^10^. Among the various detection methods available, qRT-PCR is the preferred method for gene detection owing to its high sensitivity, strong specificity, and convenient operation^11–13^. Currently, qRT-PCR based on stem-loop and poly(A) methods, which use primers designed specifically for this purpose, is commonly used for miRNA detection. The stem-loop method^14^ offers greater specificity and sensitivity compared to the poly(A) method^15–17^. The selection of miRNA-related reference genes varies among different cultivars, and lilies encompass multiple varieties. For instance, in a study on *Lilium pumilum* DC. Fisch. For somatic embryos, the reference genes selected were lpu-miR159a and the F-box family protein (FP)^18^. In *Lilium* × *formolongi*, 5S RNA and EF were selected as reference genes^19^. In the OT (Oriental × Trumpet) hybrid ‘Robina’, U4 was reported as the reference gene^20^. Among the bicolor cultivars of Asiatic hybrid lily, namely, Lollypop, Cancún, Vermeer, Sugar Love, and Enjoys, as well asnthree full-color cultivars of the species, namely, Côte d’Azur, Vivaldi, and Montreux, U6 was identified as the reference gene^21^. In *Lilium leichtlinii* var. *maximowiczii*, 18S rRNA was reported as the reference gene^5^. *Lilium henryi* Baker is a species endemic to China and located primarily in regions such as Hubei and Guizhou. This species has characteristic orange flowers, which confer high ornamental value. The bulbs of *Lilium henryi* Baker are used for human consumption and also in traditional medicine^22^. The species exhibits strong adaptability^23^ and excellent overall resistance. It is the only wild species in the Lilium genus with a vining growth habit, owing to which it is considered an important breeding material in horticulture. The species is at the state of NT (Near Threatened) according the IUCN standard (http://www.iplant.cn/rep/prot/Lilium%20henryi). For the above reasons, a detailed investigation of *Lilium henryi* Baker is of paramount importance. In the same context, the selection of miRNA reference genes and the establishment of a qRT-PCR detection system for *Lilium henryi* Baker are imperative.

Our research group pioneered miRNA omics studies related to flower color traits in *Lilium henryi* Baker (yet to be published). A small RNA sequencing of *Lilium henryi* Baker was conducted and revealed numerous miRNA sequences. Among those sequences, ten miRNAs with relatively stable expression were screened out and used as candidate reference genes. In addition, two commonly used reference genes were also selected. The stability of the candidate reference genes was analyzed using different software tools, such as geNorm^24^, NormFinder^25^, and BestKeeper^26^. The objective of this study was to identify suitable miRNA reference genes for different growth and development stages and various tissues to provide appropriate internal reference choices for accurate quantification of miRNA gene expression levels during the growth and development of *Lilium henryi* Baker.

## Materials and methods

### Experimental materials

**Figure 1:**
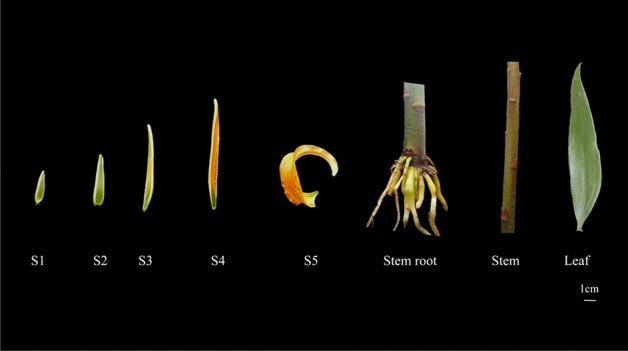
Illustration of the five stages of petal growth and development (first five images on the left) in *Lilium henryi* Baker. S1: green bud stage, S2: bud stage, S3: coloring stage, S4: early blooming stage, S5: full blooming stage. Illustration of the stem-root, stem, and leaf tissues (three images on the right) of *Lilium henryi* Baker.

*Lilium henryi* Baker was used as the experimental material in the present study (Fig. 1), Fig. 1 generate using Illustrator 2021 software. The plants of this species were cultivated in a greenhouse at the Beijing Academy of Agriculture and Forestry Sciences. Samples were collected at different stages of flower development (S1: green bud stage; S2: bud stage; S3: coloring stage; S4: early blooming stage; and S5: full blooming stage) and also from the various tissues (petals at the full blooming stage, stem-root, stem, and leaves). Three biological replicates were performed for each group of samples. After collection, the samples were rapidly frozen in liquid nitrogen and stored at − 80 °C until use.

### Experimental methods

#### Extraction and reverse transcription of the miRNAs

The required miRNAs (from the petals of *Lilium henryi* Baker at the five stages of development and also from the stem-root, stem, and leaf tissues of the species) were extracted using the miRcute miRNA Isolation Kit (Tiangen Biotech Co.). The integrity of the extracted miRNA was assessed using agarose gel electrophoresis, and the purity and concentration of the miRNA were determined using a NanoDrop 2000. After quality control analysis, the miRNAs were reverse transcribed using the miRNA 1st Strand cDNA Synthesis Kit (using the stem-loop method) from Nanjing Vazyme Biotech Co. Ltd., following the manufacturer’s instructions. First-strand cDNA synthesis was performed in a reaction volume of 20 µL, which contained 0.5 µg of the miRNA to be reverse transcribed in an RNase-free centrifuge tube. In this process, the genomic DNA was first removed from the miRNA sample by heating at 42 °C for 2 min. Afterward, the first-strand cDNA synthesis reaction was conducted using the following temperature program: 25 °C for 5 min, 50 °C for 15 min, and finally, 85 °C for 5 min.

#### Screening of the candidate reference genes and primer design

Using our research group’s published small RNA sequencing data for *Lilium henryi* Baker (PRJNA1014586), ten miRNAs with relatively stable expression were selected as candidate reference genes. In addition, commonly used reference genes for plant miRNA qPCR were also selected, along with four miRNAs for validation. The sequences of primers used for all of the above analyses are provided in Table 1. These sequences were synthesized at Beijing Tsingke Biotech Co. Ltd.

**Table 1:** Primer information for the 12 candidate reference genes and 4 validation genes.

Gene symbol	Gene name	Primer sequence (5′-3′)	Length (nt)
U6	Small nuclear RNA	F:CGGGGACATCCGATAAAATTGGAACG	26
		R:CGATTTGTGCGTGTCATCCTTGC	23
18S	18S rRNA	F:CCTGAGACGGCTACCACAT	19
		R:CACCAGACTTGCCCTCCA	18
novel_1	microRNA	F:GCGTTCCACGGCTTTCTT	18
		R:GTCGTATCCAGTGCAGGGTCCGAGGTATTCGCACTGGATACGACCAGTTC	50
novel_20	microRNA	F:CGCGTTTTCTGTCCGGTCTT	20
		R:GTCGTATCCAGTGCAGGGTCCGAGGTATTCGCACTGGATACGACAGTAGA	50
novel_69	microRNA	F:GCGTTTTTGGGCCAACTG	18
		R:GTCGTATCCAGTGCAGGGTCCGAGGTATTCGCACTGGATACGACACACAT	50
novel_77	microRNA	F:CGCGTTTGGATTGAAGGGA	19
		R:GTCGTATCCAGTGCAGGGTCCGAGGTATTCGCACTGGATACGACTAGAGC	50
osa-miR159f.	microRNA	F:GCGCTTGGATTGAAGGGA	18
		R:GTCGTATCCAGTGCAGGGTCCGAGGTATTCGCACTGGATACGACTAGAGC	50
osa-miR160a-5p	microRNA	F:CGTGCCTGGCTCCCTGT	17
		R:GTCGTATCCAGTGCAGGGTCCGAGGTATTCGCACTGGATACGACTGGCAT	50
osa-miR166a-3p	microRNA	F:GCGTCGGACCAGGCTTCA	18
		R:GTCGTATCCAGTGCAGGGTCCGAGGTATTCGCACTGGATACGACGGGGAA	50
osa-miR166g-3p	microRNA	F:GCGTCGGACCAGGCTTCA	18
		R:GTCGTATCCAGTGCAGGGTCCGAGGTATTCGCACTGGATACGACGAGGAA	50
osa-miR166m	microRNA	F:GCGTCGGACCAGGCTTCA	18
		R:GTCGTATCCAGTGCAGGGTCCGAGGTATTCGCACTGGATACGACAGGGAA	50
osa-miR167d-5p	microRNA	F:GCGTGAAGCTGCCAGCAT	18
		R:GTCGTATCCAGTGCAGGGTCCGAGGTATTCGCACTGGATACGACCAGATC	50
Target genes			
osa-miR156a	microRNA	F:CGCGCGTGACAGAAGAGAGT	20
		R:GTCGTATCCAGTGCAGGGTCCGAGGTATTCGCACTGGATACGACGTGCTC	50
osa-miR395b	microRNA	F:GCGGTGAAGTGTTTGGGG	18
		R:GTCGTATCCAGTGCAGGGTCCGAGGTATTCGCACTGGATACGACGAGTTC	50
osa-miR396a-3p	microRNA	F:GCGCGGTTCAATAAAGCTG	19
		R:GTCGTATCCAGTGCAGGGTCCGAGGTATTCGCACTGGATACGACTTCCCA	50
osa-miR396a-5p	microRNA	F:CGCGTTCCACAGCTTTCTT	19
		R:GTCGTATCCAGTGCAGGGTCCGAGGTATTCGCACTGGATACGACCAGTTC	50

#### qRT-PCR analysis of the candidate reference genes

A 50 µL reaction mixture, which contained 5 µL of the cDNA template, 2 µL each (10 µM) of forward and reverse primers, 25 µL of 2 × miRNA Universal SYBR qPCR Master Mix, and 16 µL of ddH_2_O, was prepared according to the instructions of the Vazyme miRNA Universal SYBR qPCR Master Mix Kit. Each reaction was performed in triplicate. The PCR was conducted using the following program: initial denaturation at 95 °C for 5 min, followed by 40 cycles of denaturation at 95 °C for 10 s, followed by annealing at 60 °C for 30 s, and a final extension step at 72 °C for 15 s. A melting curve analysis was conducted using the following temperature program: 95 °C for 15 s, 60 °C for 60 s, and 95 °C for 15 s. Each reaction system was run in triplicate.

The amplification efficiency of the primers was determined by generating a standard curve using a fivefold dilution series of the cDNA template in water (v:v) –at 1:4, 1:24, 1:124, 1:624, and 1:3124. After each dilution, qRT-PCR was performed for each primer pair to obtain the Cq values and generate the standard curve. The amplification efficiency (E) and the R^2^ value were calculated, and the correction equation was determined to be: E = (5 ^−1/slope^ − 1) × 100%^27,28^.

### Data processing and analysis

The data obtained was processed and analyzed using the geNorm, NormFinder, and BestKeeper software programs, the Delta CT method^29^, and the RefFinder online tool^30^. These algorithms were subsequently used to analyze the expression stability of the candidate reference genes during the five different stages of petal growth and development and in the various tissues of the species, including petals at the full blooming stage, stem-root, stem, and leaves.

## Results

### Analysis of the specificity and amplification efficiency of the primers

**Figure 2:**
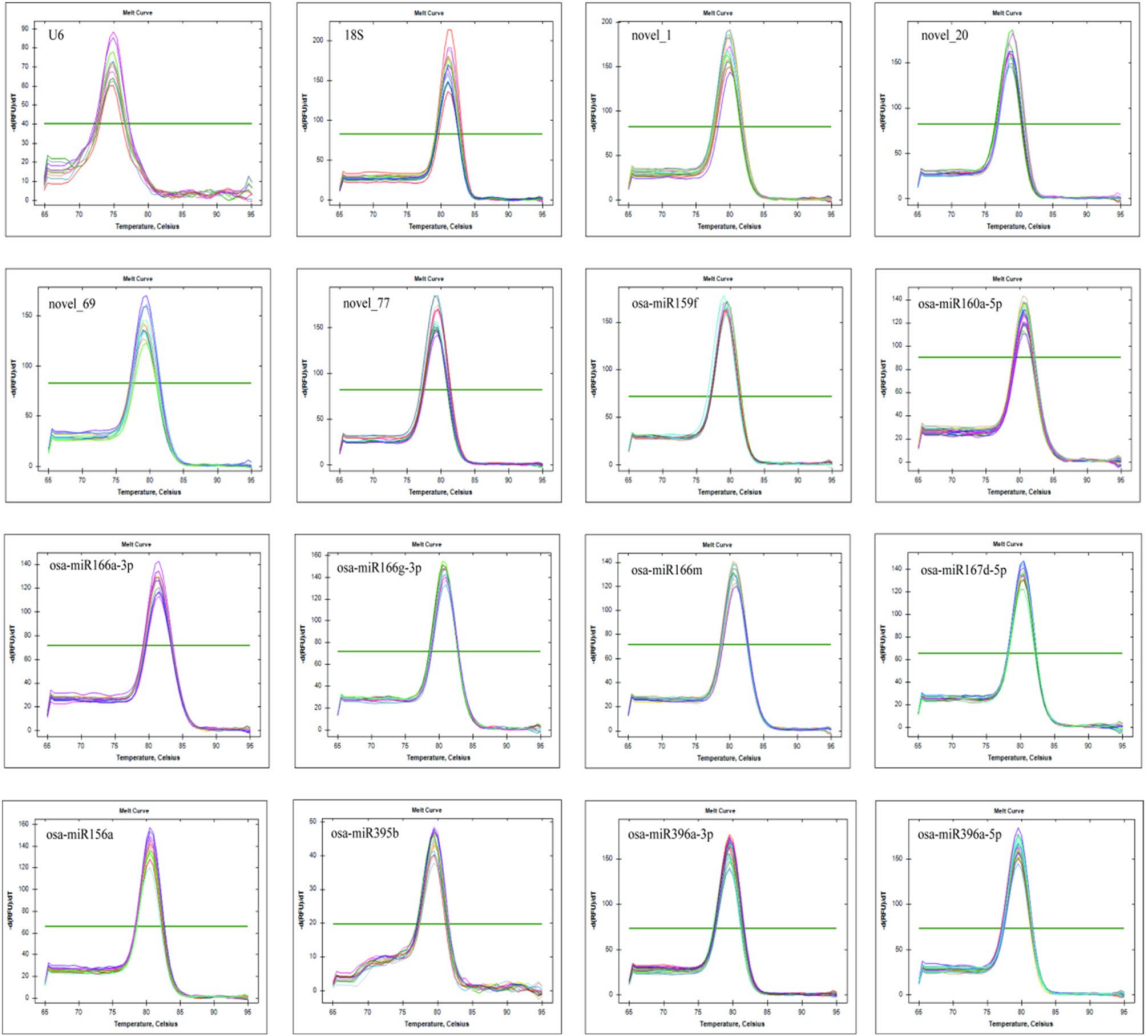
Melting curves for the qRT- PCR amplification products of the 12 candidate reference genes and 4 validation genes.

qRT-PCR analysis was performed using first-strand cDNA synthesized from petal, stem-root, stem, and leaf samples collected at five different stages of growth and development. The resulting melting curves of all 12 candidate reference genes and those of the 4 validation genes presented a single distinct peak (Fig. 2), indicating the specificity of the primers. The replicates of each sample exhibited good consistency, further confirming the specificity of the primers. The amplification efficiency of each primer was determined based on the standard curves (Table 2). All primers exhibited good specificity and amplification efficiency and met the experimental standards for qRT- PCR. Therefore, these primers were used in subsequent experiments.

**Table 2:** Description of the amplicon characteristics for each candidate reference gene.

Gene symbol	PCR Efficiency (E%)	Regression coefficient (R^2^)
U6	1.080	0.994
18S	1.007	0.995
novel_1	0.946	0.991
novel_20	0.982	0.994
novel_69	1.057	0.992
novel_77	1.124	0.979
osa-miR159f.	1.121	0.989
osa-miR160a-5p	0.984	0.987
osa-miR166a-3p	1.075	0.994
osa-miR166g-3p	1.041	0.993
osa-miR166 m	0.965	0.994
osa-miR167d-5p	1.074	0.967
osa-miR156a	1.009	0.995
osa-miR395b	1.056	0.996
osa-miR396a-3p	1.008	0.987
osa-miR396a-5p	1.034	0.993

### Expression abundance of the candidate reference genes

**Figure 3:**
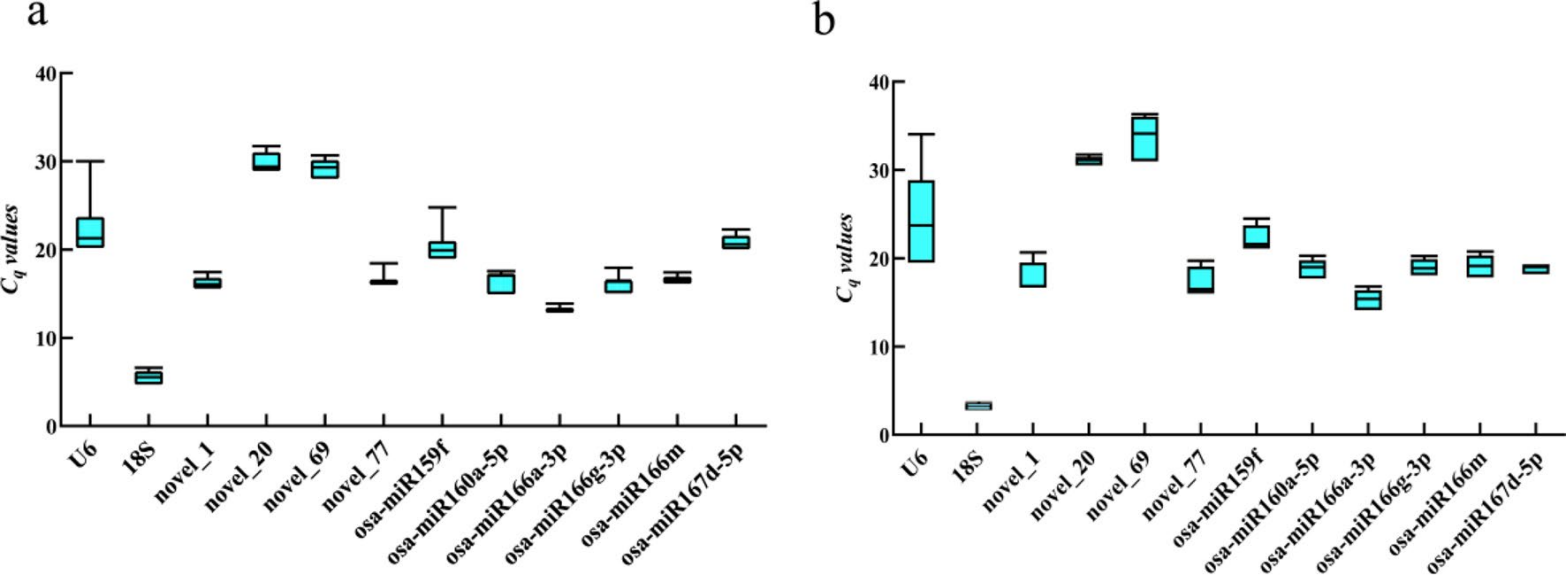
Cq values of the 12 evaluated candidate miRNA reference genes. (**a**) The Cq values for the different flowering periods. (**b**) The Cq values for different tissues.

The Cq values of the 12 candidate reference genes at different flowering stages and from various tissues of *Lilium henryi* Baker were summarized to evaluate the stability of their expression at the transcriptional level (Fig. 2). Specifically, 18S in the petals of *Lilium henryi* Baker at different developmental stages presented the lowest Cq value of 3.27, while novel_20 presented the highest Cq value of 31.37 (Fig. 3a). Among the different tissues of *Lilium henryi* Baker, the lowest Cq value of 2.76 was again obtained for 18S, while novel_69 presented the highest Cq value of 36.34 (Fig. 3b).

### Stability analysis of the candidate reference genes

#### The geNorm analysis

**Figure 4:**
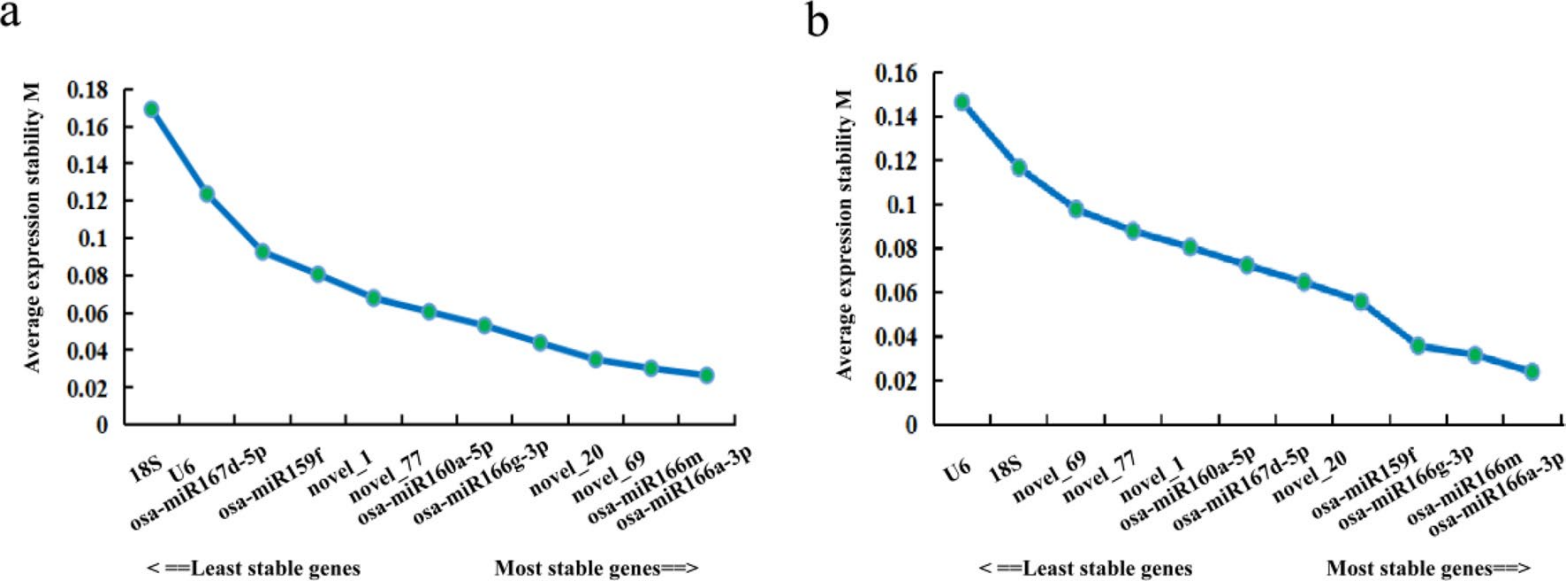
Results of geNorm analysis of the expression stability of the candidate reference genes. (**a**) The results of geNorm analysis of the expression stability of the candidate reference genes in the petals at different developmental stages. (**b**) The results of geNorm analysis of the expression stability of the candidate reference genes in the various tissues of the species.

geNorm software was used to caculate calculates the pairwise variation and average expression stability value (M value) among all the candidate genes. In each iteration, the gene with the highest M value is excluded until the most stable gene combination is obtained.The results of the geNorm analysis revealed osa-miR166a-3p and osa-miR166m as the most stable miRNA reference genes in the petals at different developmental stages of *Lilium henryi* Baker as well as in the different tissues of *Lilium henryi* Baker (Fig. 4).

Among the petals at different developmental stages in *Lilium henryi* Baker, all had M- values significantly lower than 1.5, indicating relatively stable expression levels. The three most stable miRNA internal controls were osa-miR166a-3p, osa-miR166m, and novel_69 (Fig. 4a). The stability of the candidate miRNA internal reference genes in the different tissues of *Lilium henryi* Baker indicated relatively stable expression levels. The three most stable miRNA internal controls were osa-miR166a-3p, osa-miR166m, and osa-miR166g-3p. However, the expression stabilities of U6 and 18S were the lowest among the 12 genes (Fig. 4b).

**Figure 5:**
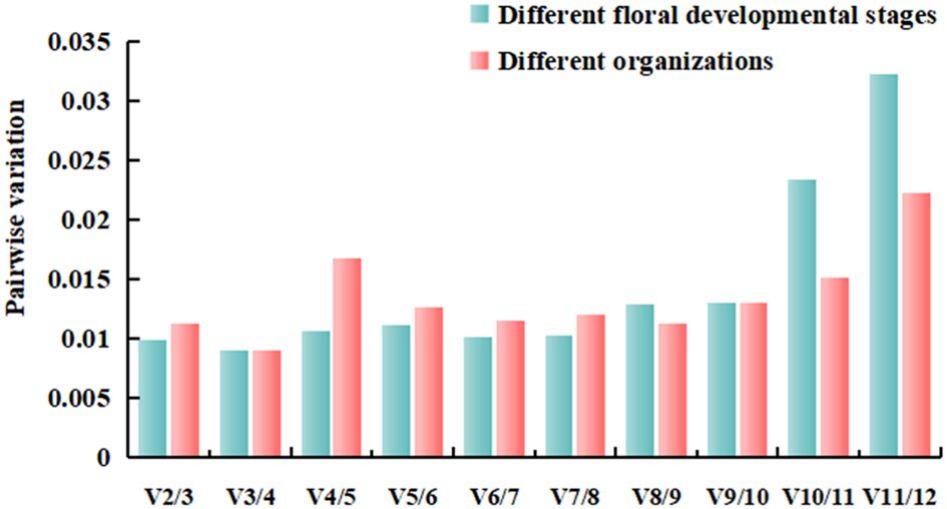
The pairwise variations in the references.

Paolacci et al.^31^ suggested using multiple internal reference genes for data correction and thereby obtaining accurate results. Therefore, it is considered necessary to combine pairwise variation to predict the optimal number of internal reference genes by calculating the value of Vn/n + 1. Accordingly, when Vn/Vn + 1 < 0.15, the optimal number of reference genes is n. When Vn/Vn + 1 > 0.15, the optimal number of reference genes is n + 1^32^. In the present study, all the obtained Vn/Vn + 1 values were less than 0.15 (Fig. 5), of which 2 was the optimal number of internal reference genes for this experiment. Accordingly, by considering both M and V values, the suitable internal reference genes for the petals at the different developmental stages of *Lilium henryi* Baker and also for the various tissues of the species were osa-miR166a-3p and osa-miR166m.

#### NormFinder analysis

**Figure 6:**
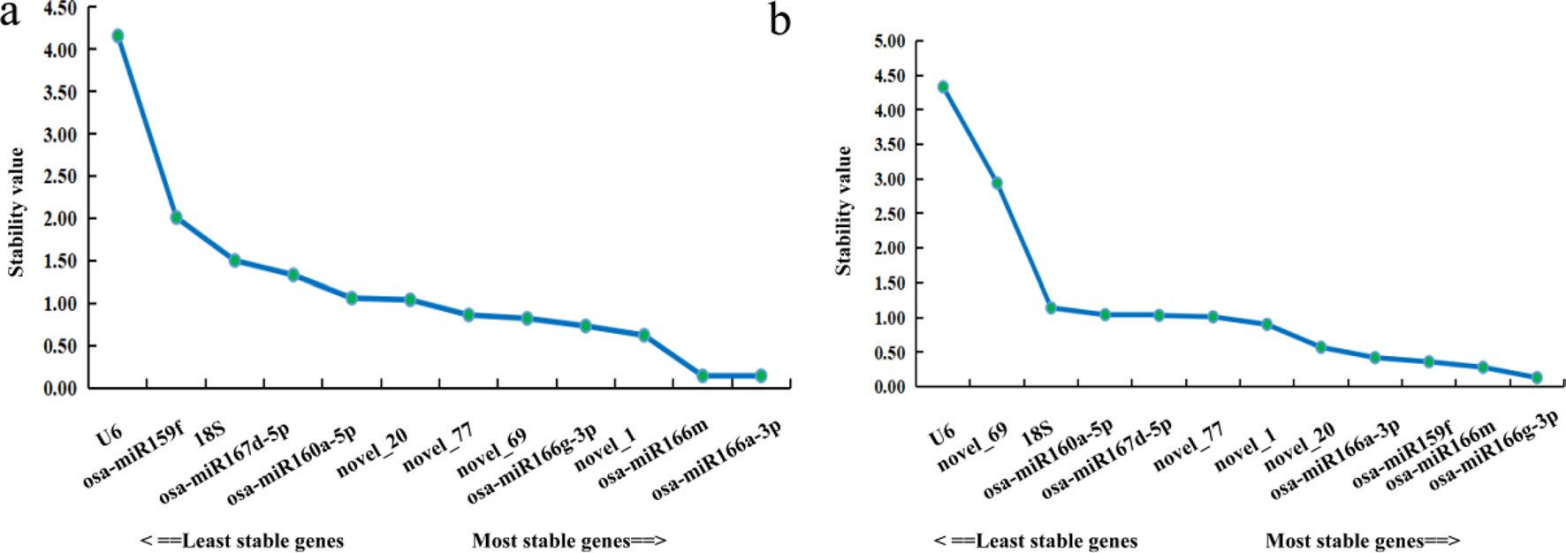
Results of NormFinder analysis of the expression stability of candidate reference genes. (**a**) The results of NormFinder analysis of the expression stability of candidate reference genes in petals at ifferent developmental stages of the species. (**b**) The results of the NormFinder analysis of the expression stability of candidate reference genes in the various tissues of the species.

NormFinder ranks the stability of genes based on their expression stability values, with smaller stability values indicating a further stable gene expression^25^.The results of NormFinder analysis revealed that in petals at different developmental stages of *Lilium henryi* Baker (Fig. 6a). The most stable miRNA reference gene with a stability value of 0.1 was osa-miR166m, and osa-miR166a-3p was also relatively stable with a stability value of 0.14. According to this analysis, the least stable reference genes were U6 and osa-miR159f. In the different tissues of *Lilium henryi* Baker were used (Fig. 6b). The most stable miRNA reference gene with a stability value of 0.13 was osa-miR166g-3p, and osa-miR166m was also relatively stable, with a stability value of 0.28. The highest expression stability values were obtained for U6 and novel_69, indicating that their expression stability was the poorest among all the candidate genes, therefore, these genes were not suitable for use as reference genes.

#### BestKeeper analysis

BestKeeper software primarily evaluates the stability of candidate reference genes by comparing the standard deviation (SD) and the coefficient of variation (CV) of their CT values. Smaller CV and SD values indicate greater stability of the candidate reference gene and its suitability as an internal reference.

**Figure 7:**
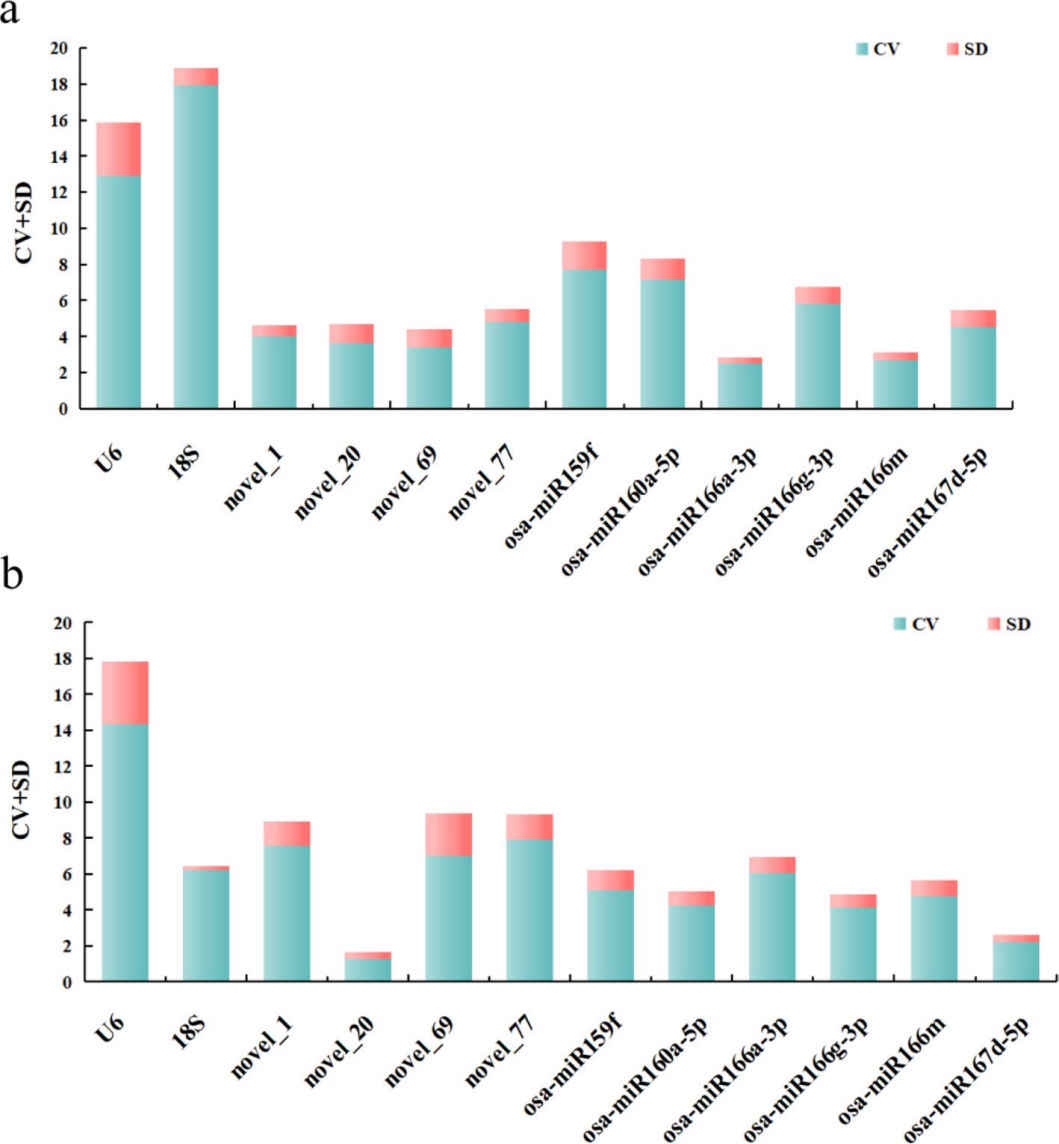
Analysis of the expression stability of the candidate internal reference genes using BestKeeper software. (**a**) BestKeeper analysis results for the expression stability of internal reference genes in petals at different developmental stages. (**b**) BestKeeper analysis results for the expression stability of internal reference genes in different tissues of the species.

In general, an SD value of less than 1 is considered indicative of stable gene expression. BestKeeper analysis of the petals at different developmental stages revealed 8 genes with an SD < 1, while 4 genes had an SD above 1.The 3 most stable miRNAs were osa-miR166a-3p, osa-miR166m, and novel_1, with osa-miR166a-3p exhibiting the smallest standard deviation and coefficient of variation values, which indicated that it was the most stable internal reference gene. U6 exhibited the lowest stability, with the largest standard deviation and coefficient of variation (Fig. 7a). BestKeeper analysis results of the various tissues revealed 7 genes with SDs < 1, and the smallest SD value of 0.2 was obtained for 18S. Only 5 genes had an SD above 1. The order of stability of the miRNA reference g genes was as follows: 18S > novel_20 > osa-miR167d-5p > osa-miR166g-3p > osa-miR160a-5p > osa-miR166m > osa-miR166a-3p > osa-miR159f. > novel_1 > novel_77 > novel_69 > U6. The smallest sum of thestandard deviation and coefficient of variation values was obtained for novel_20, while U6 exhibited the lowest stability (Fig. 7b).

#### Delta CT analysis

The Delta CT method was used to selects the optimal candidate internal reference gene based on the mean standard deviation (mean SD) value^26^. A lower mean SDobtained for an internal reference gene indicates a higher expression stability.

**Figure 8:**
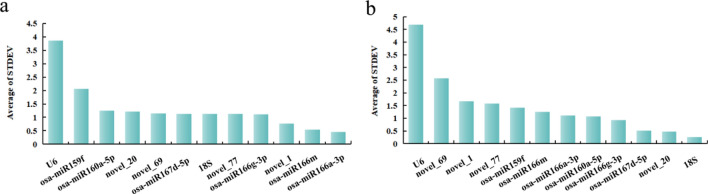
Expression stability analysis of candidate internal reference genes using the Delta CT method. (**a**) The results of the expression stability analysis of candidate internal reference genes in the petals at different developmental stages of the species using the Delta CT method. (**b**) The results of the expression stability analysis of candidate internal reference genes in the different tissues of the species using the Delta CT method.

Delta CT analysis of petals at different developmental stages. The mean SD values of osa-miR166a-3p, osa-miR166m, and novel_1 were relatively low, indicating their good expression stability across all the samples. The most stable internal reference gene was osa-miR166a-3p, while U6 presented the largest mean SD value, indicating that U6 has the poorest stability (Fig. 8a). Among the different tissues of *Lilium henryi* Baker, 18S, novel_20, and osa-miR167d-5p presented relatively small mean SD values, indicating good expression stability across all the samples. U6 presented the largest mean SD, indicating that it had the poorest stability (Fig. 8b).

#### RefFinder analysis

**Figure 9:**
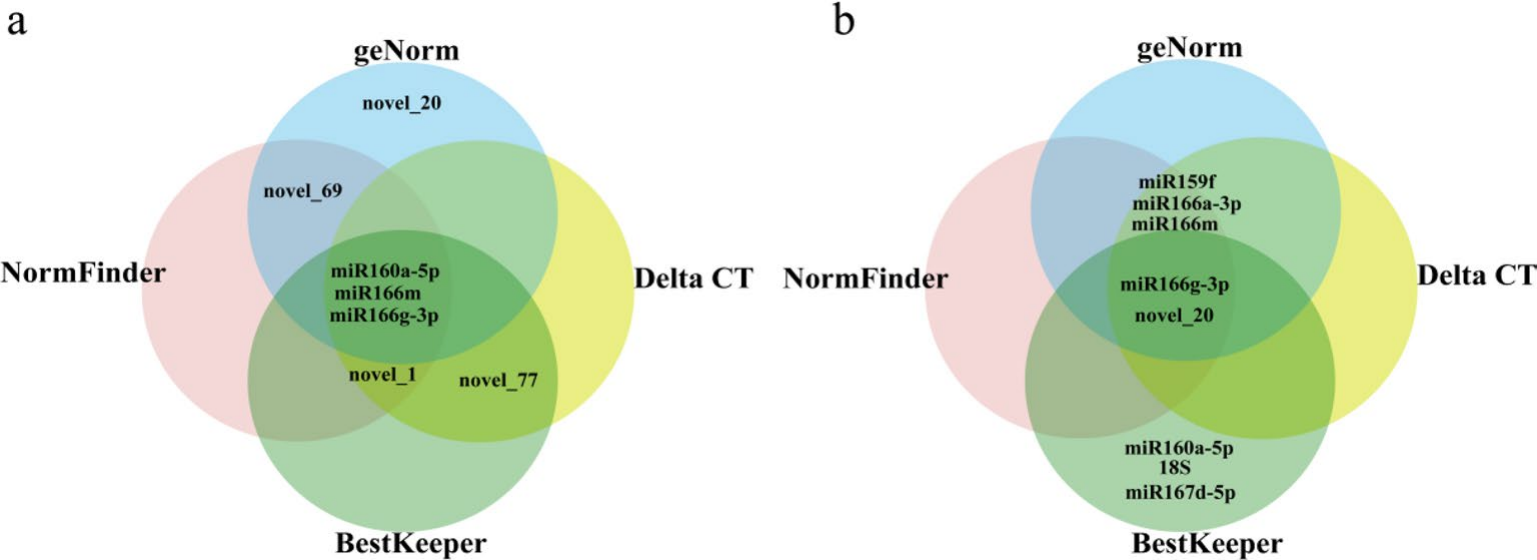
The five most stable reference genes selected via geNorm, NormFinder, BestKeeper, and Delta CT analyses. (**a**) Venn diagram of the expression stability of candidate reference genes in petals at different developmental stages. (**b**) Venn diagram of the expression stability of candidate reference genes in the different tissues of the species.

According to the ranks assigned to the genes based on each of the four programs, RefFinder assigns an appropriate weight to each gene and calculates the geometric mean of these weights. A resultant lower value indicates higher stability of the candidate internal reference gene under the given experimental conditions. Figure 9 shows the Venn diagram of the 5 most stable internal reference genes identified based on the geNorm, NormFinder, BestKeeper, and Delta CT analyses.

**Figure 10:**
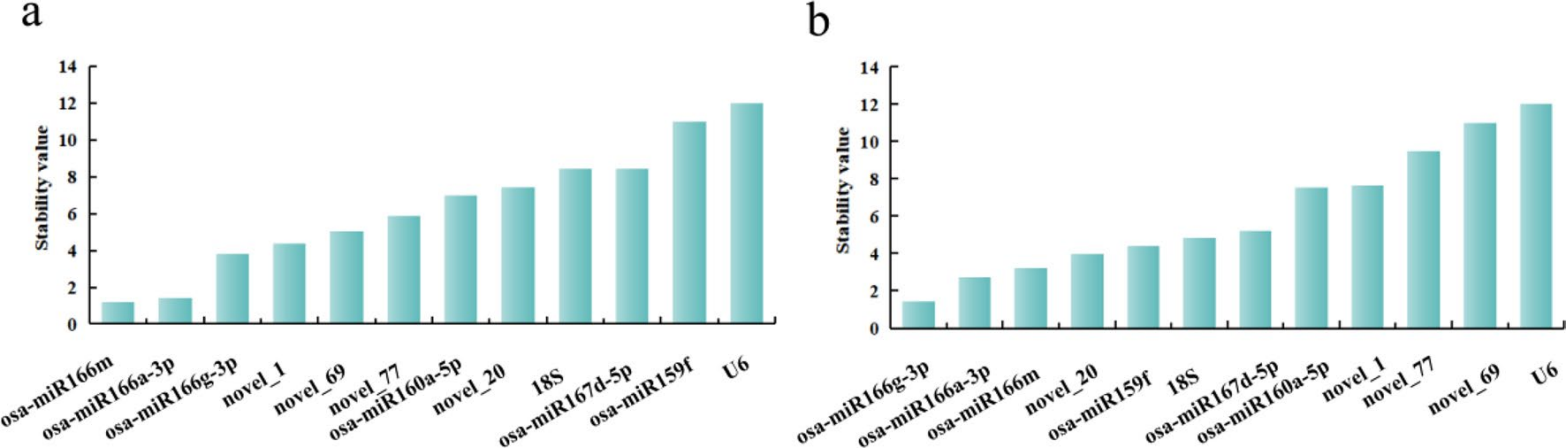
Results of RefFinder analysis of the expression stability of candidate reference genes. (**a**) The results of RefFinder analysis of the expression stability of candidate reference genes in petals at different developmental stages. (**b**) The results of the RefFinder analysis of the expression stability of candidate reference genes in the different tissues of the species.

The results of the comprehensive analysis conducted using RefFinder revealed that in *Lilium henryi* Baker petals at different developmental stages (Fig. 10a). According to the results of the analysis based on the five evaluation methods, osa-miR166m, and osa-miR166a-3p exhibited relatively stable expression, with corresponding stability values of 1.19 and 1.41, respectively. U6 exhibited the least stable expression. According to these results of the geNorm analysis, the optimal number of internal reference genes to be introduced in the experiment was 2: osa-miR166m and osa-miR166a-3p. The different tissues of *Lilium henryi* Baker were used (Fig. 10b). According to the results of the analysis based on the five evaluation methods, osa-miR166g-3p and osa-miR166a-3p exhibited relatively stable expression, with corresponding stability values of 1.41 and 2.74, respectively. U6 exhibited the least stable expression. According to the results of geNorm analysis, the optimal number of internal reference genes to be introduced in the experiment was again 2: osa-miR166g-3p and osa-miR166a-3p. According to all the algorithms used in the present study, in *Lilium henryi* Baker, the miR166 family members, including osa-miR166a-3p, osa-miR166g-3p, and osa-miR166m, consistently ranked high in stability. Therefore, it was inferred that the miR166 family genes could serve as suitable candidates for use as internal reference genes in both petals at the different developmental stages of *Lilium henryi* Baker and in the different tissues of the species.

#### Validation of genes

**Figure 11:**
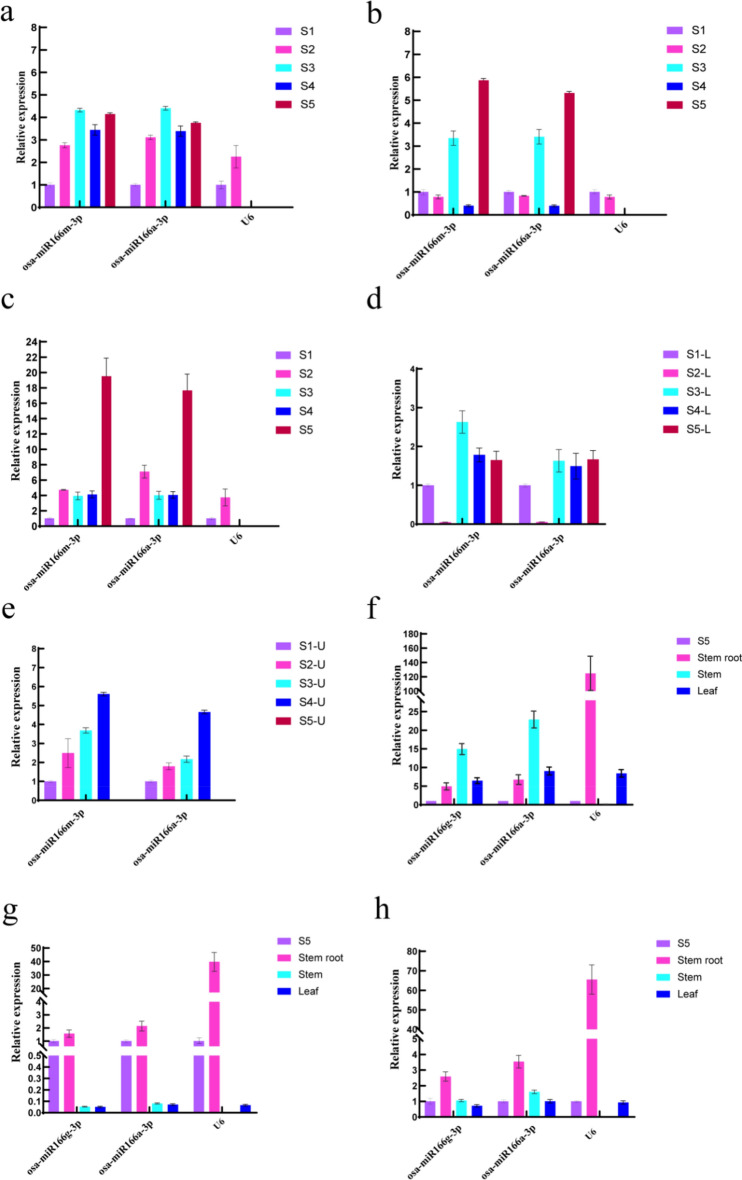
The expression profiles of the three validated genes in petals at different developmental stages and in the various tissues of the species. (**a–c**) The expression patterns of osa-miR156a, osa-miR396a-3p, and osa-miR396a-5p, in petals at different developmental stages. (**d, e**) The expression patterns of osa-miR395b, at different developmental stages and in the lower and upper parts of petals. (**f–h**) The expression patterns of osa-miR156a, osa-miR396a-3p, and osa-miR396a-5p in different tissues.

According to previous studies, miR156 plays an important role in regulating flowering time, lateral root development, and anthocyanidin synthesis^33–35^. miR396 is also widely involved in the regulation of growth and development in plant petals, leaves, and other parts^36,37^.Therefore, the expression levels of miR156 and miR396 were verified. Among the petals at the different developmental stages of the species, when the most stable reference genes were used, osa-miR166m and osa-miR166a-3p, the expression patterns of the target gene osa-miR156a were consistent (Fig. 11a). This gene exhibited the lowest expression level during the green bud stage, reached its maximum expression level during the coloring stage as the petals developed, then again exhibited a decreased expression level during the initial opening stage, and finally, a slight increase. However, for the least stable reference gene, U6, the expression level of osa-miR156a was quite low during the coloring, initial opening, and full bloom stages.

Similarly, the expression pattern of the target gene osa-miR396a-3p was consistent (Fig. 11b) in the different developmental stages of petals, with slight decreases during the bud stage, increases during the coloring stage, sharp decreases during the initial opening stage, and a maximum expression level during the full bloom stage. However, when U6, the least stable gene, was used as the reference gene, the expression pattern of osa-miR396a-3p varied and was opposite to that observed when osa-miR166m and osa-miR166a-3p were used as reference genes. The expression pattern of the target gene osa-miR396a-5p was consistent (Fig. 11c) among the different developmental stages of petals, with the maximum expression occuring during the full bloom stage. Therefore, osa-miR156a and osa-miR396a may have regulatory effects on the growth and development of petals, especially during the blooming stage.

Due to the gradual formation of spots in the lower part of petals during development and because the main component of the spots is anthocyanins, some studies suggest that osa-miR395b is a key gene related to anthocyanin synthesis^38^. Therefore, osa-miR166m and osa-miR166a-3p should be used as reference genes during the five stages of petal development to determine the expression of osa-miR395b in the lower and upper parts of petals o further verify the reliability of osa-miR166m and osa-miR166a-3p as internal reference genes.The expression trend of osa-miR395b was consistent when osa-miR166m and osa-miR166a-3p were used as internal reference genes. The expression level of osa-miR395b was extremely low in the lower part of the bud stage (Fig. 11d) and then significantly increased during the coloring stage. However, in the upper part of the petals (Fig. 11e), the expression level of osa-miR395b gradually increased during the green bud stage, bud stage, coloring stage, and early bloom stage, while the expression level was extremely low in the full blooming stage. This further proves the accuracy of osa-miR166m and osa-miR166a-3p as internal reference genes, whether for the entire petal or for different parts of the petal.

Among the different tissues, when the most stable reference genes, osa-miR166g-3p and osa-miR166a-3p, were used, the expression pattern of the target gene osa-miR156a remained consistent (Fig. 11f), with the lowest expression level in the petals observed during the full bloom stage and the highest expression level observed in the stem. Therefore, osa-miR156a may be involved in the regulation of stem growth.However, when the least stable reference gene U6 was used, the expression pattern was different, with the highest expression level observed in the stem and the lowest expression level observed in the petals at the full blooming stage. Similarly, the expression patterns of the target gene osa-miR396a-3p (Fig. 11g) in the petals and the stem were consistent, with relatively higher expression levels observed, while lower expression levels of this gene were observed in the stem and leaves. However, when the least stable reference gene U6 was used, the expression level of osa-miR396a-3p was extremely high in the stem. The expression pattern of the target gene osa-miR396a-5p (Fig. 11h) also remained consistent, with the highest expression level observed in the stem, while relatively lower expression levels were observed in the stem, leaves, and petals at the full bloom stage. Therefore, osa-miR396a-3p and osa-miR396a-5p may be involved in the growth and development of stem rooting. However, when the least stable reference gene U6 was used, significant differences in the expression of osa-miR396a-5p were observed among the different tissues, with the expression level in the stem approaching 0.

## Discussion

The application of qRT-PCR for the quantitative analysis of the expression profiles of key genes is a fundamental and widely employed strategy for deciphering the mechanisms of plant growth and development. The selection of reference genes, particularly the internal control genes, has a crucial influence on the accuracy of gene expression profiles^39,40^. The screening and validation of miRNA reference genes have been reported for several plants, including *Brassica napu*s^41^, grapevine (*Vitis vinifera* L. ‘Muscat Hamburg’)^42^, and rice (*Oryza sativa*)^43^. In poplar, U6 was identified as the most suitable reference gene for miRNA qRT-PCR experiments^44^. In sugarcane buds, miR171 and 18S rRNA were revealed as the most suitable reference genes for individual use^16^. In Chinese cedar (*Cryptomeria fortunei*), the most stable reference genes for miRNA expression were identified as novel16, cln-miR6725, novel1, and U6^45^.

The expression levels of reference genes vary significantly among different plant varieties, tissues, organs, or physiological states. For instance, in a study by Yu et al.^46^, the reference gene used in the salt stress response experiment in the soybean variety ‘Williams 82’ was gma-miR1520d. However, Liu et al.^47^ suggested that in Soybean seeds (‘Williams 82’) under different abiotic stress conditions, the most suitable combination of reference miRNAs was miR166a and miR167d in the leaf samples and miR171a and miR156a or miR167a and miR171a in the root samples.

Related research has also been conducted in the field of lilies. Zhang et al.^48^ used 5S as an internal reference gene in *Lilium* × *formolongi*. Gao et al.^49^ used 18S as an internal reference gene in *Lilium regale*. Moreover, U4 and U6 have also been used as internal reference genes in lilies^50,51^. Therefore, it was considered necessary to conduct a study on the screening of miRNA reference genes in *Lilium henryi* Baker. In this context, the present study was conducted to determine the suitable reference genes for *Lilium henryi* Baker. A total of 12 candidate genes were selected, and their performance was evaluated in different samples (flower petals at different development stages, stems, stem-roots, and leaves) of *Lilium henryi* Baker. Four commonly used algorithms (geNorm, NormFinder, BestKeeper, and Delta CT) were applied to analyze the expression stability of these 12 candidate reference genes in the petals at different developmental stages and in various tissues of the species. The objective was to evaluate and determine stable reference genes. While the top five genes selected based on the results of the different algorithms were generally similar, slight differences were observed in their stability levels, which was attributable to the different calculation methods employed by each software package. However, despite these differences, the results obtained using these different algorithms were significantly consistent in terms of the selected best reference genes. According the NormFinder analysis, the stability values of osa miR166m and osa miR66a-3p were both 0.14, indicating that osa-miR166m and osa-miR166a-3p exhibited the highest stability (Fig. 6a). To further analyze the results obtained using the four algorithms, an online analysis tool referred to as RefFinder was used. The results obtained using the geNorm, NormFinder, BestKeeper, and Delta CT methods were consistent with the results obtained using RefFinder, indicating the accuracy of the software analysis. In the case of different tissues, the results obtained using geNorm and NormFinder were generally consistent, while those obtained using BestKeeper and Delta CT presented certain differences. Similar findings were reported in previous studies and therefore, these differences are acceptable from a comprehensive perspective^52–54^.

Further analysis using RefFinder revealed results consistent with those of geNorm and NormFinder analyses. The best combination of reference genes was determined based on the comprehensive analysis conducted using RefFinder. The results indicated that suitable reference genes should be selected for different experimental samples, as not all commonly used reference genes are applicable to different tissue samples or experimental conditions. This finding was similar to that reported for *Euscaphis konishii* Hayata^10^. To further validate the accuracy of the selected reference genes, osa-miR156a, osa-miR396a-3p, and osa-miR396a-5p were selected as validation genes, owing to their important regulatory roles in plant growth and development. Moreover, during the development of lily petals, spots gradually form in the lower tissue. To investigate whether the selected internal reference genes involved in petal spot formation are suitable for miRNA transcription level analysis, the expression level of the osa-miR395b gene, which is related to anthocyanin synthesis was detected. The results confirmed the accuracy of the screened reference genes, indicating that the optimal internal reference genes osa-miR166m and osa-miR166a-3p can be used for analyzing miRNA transcription levels in different tissues of petals at different developmental stages.

Previous studies have demonstrated that U6 is highly conserved across different plant species, implying that its sequence is relatively stable among different plants. Consequently, the small nuclear RNA of U6 is widely used as an internal reference gene for miRNA studies on various plants, including tea plants^55^, rice (*Oryza sativa*)^56^, and tomato^57^. However, while U6 is commonly used as a reference gene in several plant species, differences in the plant genomic structures and regulatory mechanisms may lead to variations in the expression of U6 under certain conditions. For instance, in *Lilium* species (*Lilium pumilum* DC. Fisch. and *Lilium davidii* var. unicolor)^51^, U6 reportedly exhibited relatively lower stability ranks, while in sweet potato (*Ipomoea batatas.* L.)^58^, U6 expression was relatively unstable. The present study revealed that the commonly used reference gene U6 exhibited unstable expression during the growth and development of *Lilium henryi* Baker. On the other hand, certain miRNAs from the miR166 family exhibited relatively stable expression and were more suitable as reference genes. When miR166 is used as a reference gene, it typically includes members of its subfamily, such as miR166a, miR166b, miR166c, and miR166m. The miR166 family is a group of similar miRNAs that share the same miRNA precursor sequence or have highly conserved mature miRNA sequences. These subfamily members frequently exhibit similar expression patterns and functions. Therefore, when selecting reference genes, the entire miR166 family or its multiple subfamilies may be considered for detection. This approach allows for a further comprehensive and accurate assessment of the expression levels of the miR166 family of genes used as reference genes. Similarly, in Tamarillo (*Solanum betaceum*) callus tissue samples, miR166a was reportedly the most stable miRNA^59^.

Previous studies have screened plant internal reference genes such as EF^48^, 18S^49^, U6^50^ and Actin ^51^ during embryonic development and various stress treatment, involving relatively few tissues and organs in lily. Therefore, this study screened and validated internal reference genes involved in various flower development stages and tissue parts of *Lilium henryi* Baker. A more precise validation was conducted on the selected internal reference genes in the petals, avoiding interference from specific parts of the petals on the results of selecting the best internal reference genes. Therefore,the present study provides important information for studying the role of miRNAs in the growth and development of lilies and offers a strategy and method for screening miRNA reference genes in other plants. However, further research is warranted to validate and optimize the results obtained in the present study. Future studies could consider expanding the sample size and including samples from different developmental stages to further validate the stability and applicability of the miR166 family. Further investigations based on reference gene screening could reveal the differential expression and functions of miRNAs during the growth and development of lilies, providing a solid foundation and theoretical support for unraveling the mechanisms underlying the formation of important traits and the process of molecular breeding in lilies.

## Data Availability

The datasets generated during and/or analysed during the current study are available from the corresponding author on reasonable request.
